# Asymmetry of the temporal code for space by hippocampal place cells

**DOI:** 10.1038/s41598-017-08609-3

**Published:** 2017-08-17

**Authors:** Bryan C. Souza, Adriano B. L. Tort

**Affiliations:** Brain Institute, Federal University of Rio Grande do Norte, Natal, RN 59056-450 Brazil

## Abstract

Hippocampal place cells convey spatial information through spike frequency (“rate coding”) and spike timing relative to the theta phase (“temporal coding”). Whether rate and temporal coding are due to independent or related mechanisms has been the subject of wide debate. Here we show that the spike timing of place cells couples to theta phase before major increases in firing rate, anticipating the animal’s entrance into the classical, rate-based place field. In contrast, spikes rapidly decouple from theta as the animal leaves the place field and firing rate decreases. Therefore, temporal coding has strong asymmetry around the place field center. We further show that the dynamics of temporal coding along space evolves in three stages as the animal traverses the place field: phase coupling, sharp precession and phase decoupling. These results suggest that independent mechanisms may govern rate and temporal coding.

## Introduction

The rodent hippocampus plays a role in spatial memory and navigation^1, 2^. Some hippocampal neurons, called place cells, increase their firing rate when the animal is at a specific location of the environment, known as the ‘place field’ of the cell^3^. As the animal crosses place fields, place cells form spike sequences coordinated by the hippocampal theta rhythm (~5–12 Hz) by firing action potentials progressively coupled to earlier phases of the cycle, a phenomenon known as ‘phase precession’^4^. Place fields and phase precession are considered canonical examples of rate and temporal coding, respectively, in which the firing rate of the neuron and the exact spike timing relative to the theta cycle provide information about space^5–7^. Whether temporal and rate coding are governed by independent or related mechanisms has been widely debated^8–11^. For instance, experiments by Harris *et al*. showed that changes in firing rate predict changes in spiking theta phase, and concluded that temporal and rate coding are intrinsically related^8^. On the other hand, Huxter *et al*. showed that the theta phase of spiking correlates more with animal position than with firing rate, and suggested that place cells use two independent spatial codes^10^. More recently, by simultaneously controlling for firing rate and position within the place field, Cei *et al*. demonstrated that spiking phase depends on both firing rate (for a fixed position) and position (for a fixed firing rate)^11^, thus seemingly conciliating discrepant conclusions^8, 10^. However, here we report a key new piece of evidence showing that temporal and rate coding may dissociate to a much greater extent than previously recognized in these studies.

Since temporal coding requires the coupling of place cell spikes to theta phase^8, 10^, we revisited the rate vs temporal coding debate by investigating the spatial evolution of theta coupling strength as animals traverse the place field. We found that the spike timing of place cells couples to theta before major increases in firing rate, that is, before the animal enters the classical, rate-based place field. In contrast, theta-phase coupling rapidly ceases as the animal leaves the place field and firing rate decreases. These results reveal that temporal coding has strong asymmetry around the place field center; moreover, since the spatial receptive field of temporal coding is larger before than after the place field center, they further suggest that place cell spike timing may code more for upcoming than past positions. Interestingly, we also found that place cells do not couple to theta phase at positions distant from the place field center, and that the dynamics of temporal coding along space separates into three stages during place field traversals: phase coupling, sharp precession and phase decoupling. These findings shed new light on how place cells represent space, and suggest that temporal and rate coding may be governed by independent mechanisms.

## Results

### Rescaling space by place field length

**Figure 1:**
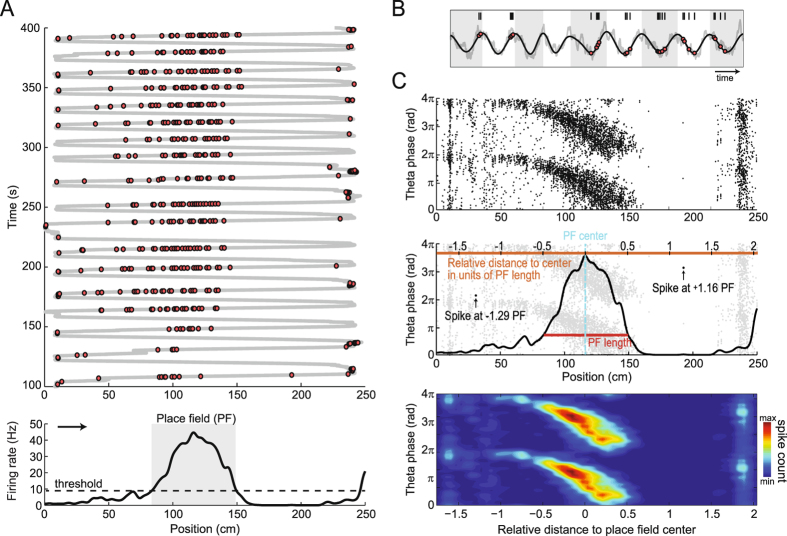
Spatial normalization by place field length. (**A**) Top, trajectory of a rat running back and forth on a linear track (gray line); red circles represent spikes of a place cell. Bottom, firing rate of the same cell as a function of position during runs to the right (arrow). The dashed line denotes the threshold used to estimate the length of the place field (gray shade). (**B**) Example of phase precession during a run to the right. Gray/white shades mark theta cycles. (**C**) Top, black dots represent all spikes of the cell in A as a function of position in cm (x-axis) and theta phase (y-axis). Middle, scheme of spatial normalization. Light gray dots represent spikes. The center of the place field was defined as the position of maximum firing rate. The orange horizontal scale on top shows the distance to the place field center in units of place field length. Arrows highlight the relative distance of two spikes. Bottom, heat map representation of spike counts per relative distance and theta phase.

We analyzed 1071 place fields from 689 place cells recorded from the hippocampal CA1 region of three rats while they ran back and forth on a linear track. Figure 1A shows spike locations for a place cell along with the animal’s trajectory (top) and the cell’s place field (bottom). As in previous work^8, 12^, we estimated place field length from a firing rate threshold, while the position of maximum firing defined the place field center. Figure 1B shows raw and theta-filtered local field potentials (LFPs) together with spike times during a place field crossing; notice the characteristic precession of spike timing within theta cycles (white/gray shades) from ascending to descending phases. Phase precession is most evident when plotting the theta phase of spiking as a function of position for multiple runs (Fig. 1C, top). To study the relation between spiking phase and space in a systematic way, we first normalized place fields to account for their variable lengths and locations. To that end, the position of each spike was expressed as the animal’s distance to the place field center divided by the place field length (Fig. 1C, middle). This procedure allowed visualization of the theta phase of spiking as a function of relative distance in units of place field length, centered at the peak activity of the place cell. Note that this procedure does not change the shape of individual place fields, and therefore does not affect firing rate skewness. Finally, as in previous work^9, 11^ we binned theta phase and space and expressed spike counts by means of heat maps (Fig. 1C, bottom).

### Theta coupling of place cells preceding place field entrance

**Figure 2:**
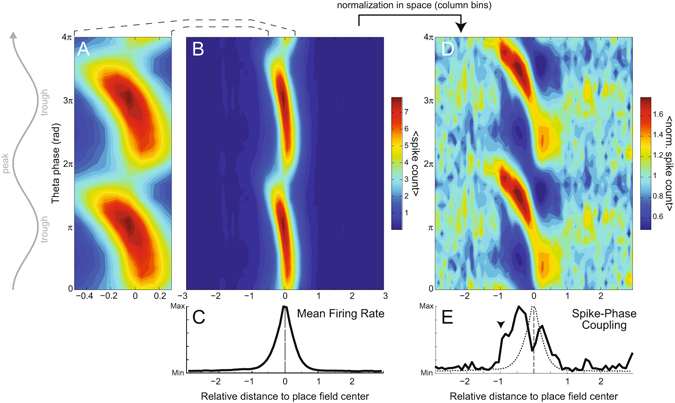
Place cells couple to theta phase before increasing their firing rate. (**A**) Average spike count per relative distance and theta phase (n = 1071 place fields), showing the characteristic phase precession (2π is the peak of theta recorded from the CA1 pyramidal cell layer). (**B**) Zoomed-out view of the data in (**A**). (**C**) Mean firing rate as a function of relative distance. (**D**) Same data as in (**B**), normalized by the mean number of spikes at each position. (**E**) Spike-phase coupling strength per relative distance (solid line); the dashed line reproduces the firing rate in (**C**). Notice that place cell spikes align to a preferred theta phase before major increases in firing rate (arrowhead).

Figure 2A,B shows the mean spike count per bin of theta phase and relative distance to the place field center (n = 1071 place fields). In these maps, the phase distribution of place cell spiking cannot be properly visualized at space bins distant from the place field center because of their much lower spike count (Fig. 2C). To circumvent this, we normalized spike counts within each space bin (a column slice) by its mean over all phases (Fig. 2D). This space-normalization allowed for assessing the phase distribution of spikes at positions away from the place field center. We found that spikes concentrate near the theta peak before the animal enters the place field, at positions where place cells exhibit no major changes in firing rate (compare Fig. 2D with B,C). Consistently, Fig. 2E shows a sharp increase in spike-phase coupling strength preceding a slower increase in firing rate; spike-phase coupling transiently decreases during the firing rate peak at the place field center, and later returns to basal levels as firing rate decreases. Notice that firing rate is comparably low before and after the place field, suggesting similar excitatory drive to place cells despite different coupling to theta at these regions.

**Figure 3:**
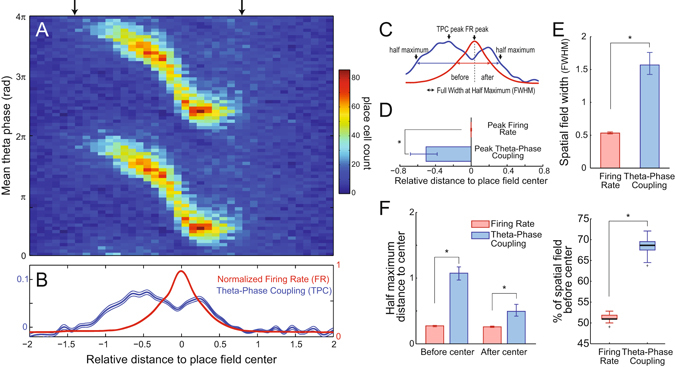
Asymmetry of the temporal code for space by hippocampal place cells. (**A**) Histogram of mean spiking phase. At each position, individual place fields contribute with one count to the phase bin in which the mean spiking of their place cell occurs. Arrows mark the region where the distribution of mean spiking phases statistically differs from the uniform distribution, indicating coordinated theta-phase coupling across cells. (**B**) Theta-phase coupling strength (TPC, blue) and mean normalized firing rate (FR, red) as functions of space. Thin lines denote 99% confidence intervals. The TPC curve was corrected by subtracting chance values (see Materials and Methods). (**C**) Scheme describing the identification of peak and half maximum values as well as of the full width at half maximum (FWHM) for TPC and FR curves. (**D**) Mean relative distance of TPC and FR peaks, showing that place cells are maximally modulated by theta phase before their peak firing rate. (**E**) Comparison of spatial field width when defined by the FWHM of FR vs TPC. (**F**) Relative distances of FR and TPC half maximum values to the place field center (left) and percentage of spatial field located before the center when defined by FR vs TPC FWHM (right). In (**D**–**F**), the error bars denote 99% confidence interval of the mean; *p < 0.01.

To assess whether the results could be due to differences in spike counts among place fields, we next calculated the mean spiking phase for each place field per bin of relative distance. Figure 3A shows the spatial histogram of mean spiking phases. In each histogram column (space bin), individual place fields contribute with a single count at the place cell’s mean spiking phase, irrespective of the number of spikes. Columns with non-uniform phase distributions indicate that place cells have similar mean spiking phase at these positions, with warmer colors denoting preferred theta phases. We next computed theta-phase coupling strength (TPC) using the distribution of mean spiking phases, and used a surrogate procedure to delimit statistically significant values (black arrows in Fig. 3A). TPC was above chance much before changes in mean normalized firing rate (FR) (Fig. 3B) and followed a time-course similar to the theta-phase coupling of pooled spikes (Fig. 2E), extending our findings from the spike to the place cell level. In fact, such a phenomenon of strong theta-phase coupling before a major increase in firing rate is also apparent in individual cells; for instance, notice that the example place cell in Fig. 1C aligns its spikes to the theta peak at ~50 cm, much before its peak firing rate at ~115 cm (see Supplementary Fig. S1 for other examples). This result also holds true when analyzing each of the three rats separately (Supplementary Fig. S2), when estimating theta phase by linear interpolation^13^ (Supplementary Fig. S3), or when analyzing the first and later spikes per theta cycle separately^14^ (Supplementary Fig. S4). Interestingly, and in contrast to previous views^15^, Figs 2E and 3B also demonstrate that the coupling of place cells to theta phase is spatially transient, which is to say that TPC – as FR – has a spatial receptive field. Moreover, they further show that different coupling strengths may be associated to the same firing rate depending on the animal’s position (e.g., compare FR and TPC at the relative distance of −1 and +1).

### Asymmetry of the temporal code for space

**Figure 4:**
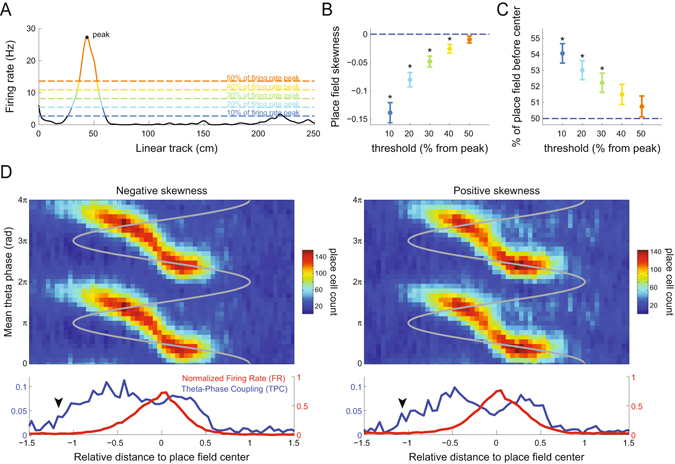
Asymmetric temporal coding irrespective of place field skewness. (**A**) Example place field. Dashed lines show different firing rate thresholds (at 10, 20, 30, 40 and 50% of the peak value) that were used to quantify place field asymmetry. (**B**) Skewness of the firing rate curve at each threshold. (**C**) Percentage of the place field located before the center (position of peak firing rate). In (**B**,**C**), data points show the mean over individual place fields; errorbars denote standard error of the mean (*p < 0.01, t-test against 0 or 50% corrected for multiple comparisons). (**D**) Panels show the same as in Fig. 3A,B, but computed using only place cells with negative (left) or positive (right) skewness at the 50% threshold.

To further characterize differences in temporal and rate coding, we computed the peak and the full width at half maximum (FWHM) of the TPC and FR curves along space (Fig. 3C). TPC peaked as rats entered the place field (mean peak distance: −0.51; CI_99_: [−0.68;−0.38]), much before the FR peak at the place field center (Fig. 3D). The FWHM of the TPC curve was significantly wider than that of the FR curve, implying that spatial fields would be larger if defined by changes in theta-phase coupling (mean FWHM for FR: 0.53; CI_99_: [0.52;0.55]; for TPC: 1.56; CI_99_: [1.42;1.79]; Fig. 3E). Moreover, while the portion of the FR curve above its half-maximum value was roughly symmetrical around the place field center (see also Fig. 4 and Supplementary Fig. S5), most of TPC FWHM occurred before the center (Fig. 3F; % FWHM before center for FR: 51.4; CI_99_: [50.0;52.8]; for TPC: 68.5; CI_99_: [65.1;71.6]). These results show that hippocampal place cells have an asymmetric temporal code for space.

It has been previously reported that rate-based place fields become skewed with experience: in familiar tracks, place cells spike more at locations preceding the location of their peak firing rate^16, 17^. Such an effect is better noticed when place field width is defined at 10% of the peak firing rate; on the other hand, place field skewness is not significantly different from 0 if computed at 50% of the peak firing rate (see Fig. 2 in ref. 16). Consistent with this, here we found that at 50% of the peak firing rate (i.e., at the FWHM), rate-based place fields were roughly symmetrical (Figs 3 and 4 and Supplementary Fig. S5), while FR skewness was significantly different from zero at 10% of the peak (−0.139 ± 0.018, p < 0.001; Fig. 4) and similar in magnitude to values reported in ref. 17 for CA1 place cells (−0.09 ± 0.04 to −0.12 ± 0.04, their Table 2). Importantly, however, the percentage of the spatial receptive field before the place field center was much larger for TPC than FR at all thresholds from the peak value (Supplementary Fig. S5), which is to say that temporal coding has much greater asymmetry around the place field center than rate coding even at thresholds in which FR is negatively skewed.

**Figure 5:**
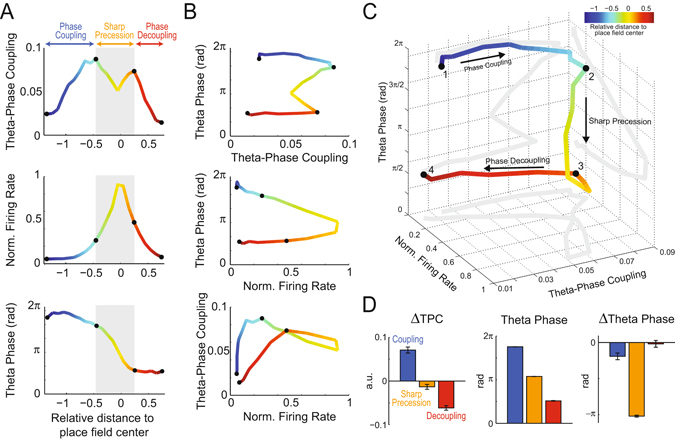
Three-stage model of space coding by place cells. (**A**) Theta-phase coupling strength (top), mean normalized firing rate (middle) and mean theta phase of spiking (bottom) per relative distance. Gray shaded area separates place cell activity along space into three stages, as labeled. (**B**) 2D plots of all pairwise combinations of the independent variables in (**A**). (**C**) 3D plot of theta-phase coupling strength, firing rate and mean spiking theta phase. Gray lines show 2D projections (same curves as in **B**). Place cells align to a preferred theta phase before major changes in firing rate and spiking phase (“Phase Coupling”; 1–2). Next, the spiking phase rapidly advances as firing rate peaks (“Sharp Precession”; 2–3). Finally, theta-phase coupling and firing rate return to basal levels while spiking occurs at the same theta phase reached after the precession (“Phase Decoupling”; 3–4). (**D**) Mean change in theta-phase coupling strength (left), mean theta phase of spiking (middle), and mean change in spiking phase (right) during each stage. In (**A**–**C**), the line color denotes the relative distance to the place field center and black dots mark stage boundaries.

**Figure 6:**
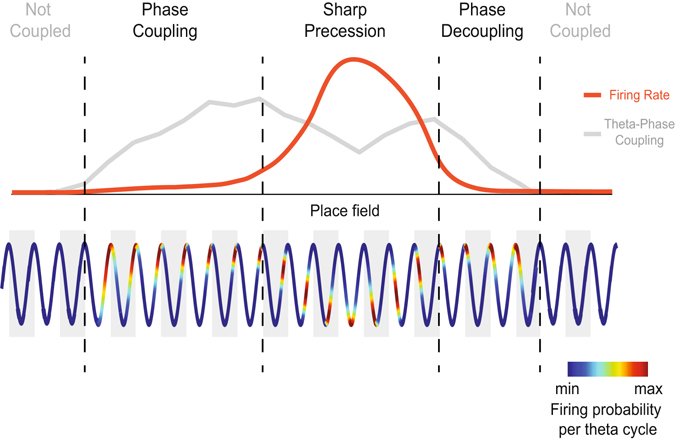
Schematic illustration of the three-stage spatial dynamics of place cell firing. The illustration depicts the changes in firing rate and in theta-phase coupling strength as the animal crosses a place field (top), as well as a schematic theta rhythm showing the probability of place cell firing within theta cycles along space (bottom). Our results suggest that the spatial dynamics of place cell activity occurs in three stages: “Phase Coupling”, when the probability of place cell firing progressively concentrates around a specific theta phase; “Sharp Precession”, when the concentrated spiking probability shifts to earlier phases of the theta cycle; and “Phase Decoupling”, when the spiking probability per theta cycle gradually returns to a uniform distribution.

Interestingly, the joint dynamics of FR and TPC revealed that space coding by place cells occurs in three stages during trajectories through the place field, and that the sharp phase precession is an intermediate stage in their temporal organization (Fig. 5; see also Supplementary Fig. S6). In the first stage, place cells align to theta while maintaining a low spiking rate; this ‘phase coupling stage’ takes place before the animal enters the place field and is characterized by a major increase in theta-phase coupling with only mild changes in spiking theta phase. Next, during the ‘sharp precession stage’, place cells display sustained levels of theta-phase coupling and spike at higher rates and at sharply varying phases of the theta cycle as the animal traverses the place field. Finally, in the third stage, place cells decouple from theta while spiking at decreasing rates at a constant theta phase (the same phase reached after precessing); this ‘phase decoupling stage’ occurs while the animal leaves the place field and is characterized by a decrease in theta-phase coupling. In Fig. 6 we provide a schematic illustration of our findings.

## Discussion

Hippocampal place cells convey spatial information through both firing rate and spike timing. In the first case, firing rate increases as the animal approaches a specific location in space known as the place field of the cell^3^ (Fig. 1A). In the second, place cells spike at varying phases of the LFP theta oscillation (~5–12 Hz), gradually advancing from the theta peak as the animal crosses the place field, a phenomenon known as phase precession^4^ (Fig. 2A). The discovery of these two neurophysiological correlates raised a debate on whether the brain uses rate and/or temporal codes for space, and whether these codes would be mechanistically related or independent^8–11^. In the present work, we revisited this debate by investigating the evolution of theta-phase coupling of spikes along space as the animal crosses the place field.

Place cells code for space by precisely timing their firing at specific phases of the theta cycle^4, 6^; therefore, the study of temporal coding intrinsically relates to the study of theta-phase coupling. However, up to now researchers have overlooked the relation between place cell spiking and theta phase at locations preceding the entrance of the animal in the cell’s place field, probably due to the low firing rate at these locations^3, 7^ (Fig. 2C). Here we performed a simple, yet novel space normalization (Fig. 2D) that allowed us to study the coupling of place cells to theta at positions of low firing. While the “banana” shape in heat-map plots of phase-precession (e.g., Fig. 2A) has been well characterized^18, 19^, to the best of our knowledge no previous study has quantified phase-locking strength to theta at positions of low firing, nor measured the dynamics of theta coupling strength along space. Together, the spatial changes of TPC, firing rate and spiking theta phase revealed a three-stage dynamics (Figs 5 and 6). The stage “phase-coupling” is defined by a large increase in TPC while place cells spike at low firing rates near the theta peak; “sharp phase precession” is defined by high firing rate and high TPC along with a substantial change of the spiking theta phase, which advances from near the theta peak to the descending phase of the cycle; the “phase decoupling” stage is defined by a major decrease in both firing rate and TPC while cells spike at a constant theta phase.

Our results show that place cell spikes are not always coupled to theta. Rather, we found that spike-phase coupling is spatially transient during crossings of the linear track, that is, place cells only couple to theta in a bounded region of space around the place field center. Both the increase in the TPC curve and its later return to zero along space are non-trivial empirical findings, for they are not explained by the definition of the metric, nor by firing rate alone (i.e. for the same firing rate, there may be different TPC levels depending on animal position). We also found that place cells align to theta phase much before they increase their firing rate, that is, much before the animal enters the rate-based place field (Figs 2E and 3B). This effect precedes the warm colors in phase-position firing maps (i.e., notice in Fig. 2 that while the characteristic “banana” shape starts at −0.4, high phase-locking to theta already occurs at −1). In contrast, we found that theta-phase decoupling and firing rate decrease happen simultaneously as the animal leaves the place field (notice in Figs 2 and 5 that this stage takes place at ~0.2, after the “banana” shape ends). These novel analyses show that hippocampal place cells display strong asymmetry between rate and temporal codes for space, and also within temporal coding itself with respect to the place field center.

It should be noted that reliably measuring spike-phase coupling requires the sampling of several spikes^20^; therefore, due to methodological constraints, the current results could only be achieved at the level of pooled spike counts or pooled place cell activity (across theta cycles and trials). In this sense, and contrasting to the sharp phase precession^21^, the stages of phase coupling and phase decoupling cannot be statistically inferred at the level of a single place cell activity in a single trial. Rather, the effect only becomes apparent when analyzing the same place cell across multiple traversals of its place field or when analyzing a population of place cells.

Interestingly, it has been previously shown that, during phase precession of grid cells, the later spikes within theta cycles have larger phase variability than the first, leading spike^14^. Moreover, phase precession slope is steeper and better correlates with position when considering only the leading spikes^14^. It is therefore possible that the phase decoupling we observed to occur upon exiting the place field is due to the high phase variability of the later spikes. However, this seems not to be the case, since we obtained similar results when analyzing the leading and later spikes per theta cycle separately (Supplementary Fig. S4).

Place cells are believed to represent the current position of the animal when firing maximally at the theta trough, while low-rate spikes at the first and second halves of the theta cycle (that is, before and after the trough) would code for past and upcoming positions, respectively^2, 18, 22, 23^. Here we found that the coupling of place cells’ spike timing to the second half of theta anticipates the entrance of the animal into the (rate-based) place field; on the other hand, place cells’ spikes rapidly decouple from the first half of the theta cycle while the animal leaves the place field. Thus, our results suggest that the representation of future positions by temporal coding reaches farther distances than the representation of past ones.

Despite considerable progress, the exact mechanism underlying phase precession remains unknown^7, 24, 25^. Here we provide additional evidence suggesting a dissociation between changes in firing rate and in theta-phase coupling. Although our results do not directly address phase precession mechanisms, they provide insights into previously proposed models. Influential theories of phase precession include dual oscillatory interference models^4^, ramp depolarization^9^, and models based on internal network dynamics^24^. Dual oscillatory models posit that phase precession is due to the interference of two oscillators of slightly different theta frequencies: one at the LFP theta frequency generated at the soma, and another at faster frequency generated at the dendrites^7, 25^. The oscillatory interference gives rise to membrane potential oscillations that change spike-phase probability across theta cycles^25^. However, the current models of oscillatory interference would not account for the different levels of phase locking at similar firing rate values observed here (i.e., when entering vs leaving the place field). The ramp depolarization model also assumes a theta-oscillating membrane potential but posits that phase precession is due to a monotonically increasing excitatory drive as the animal enters the place field^9^; this model was motivated by the negative skewness of rate-based place fields originally reported in ref. 16. In principle, such a mechanism could account for high spike-phase locking preceding entrance into the rate-based place field, assuming that low membrane depolarizations may be just enough to induce spiking locked to theta without major increases in firing rate. However, firing rate asymmetry was rather low in the present dataset (Fig. 4 and Supplementary Fig. S5; see also refs 17 and 26 for similar skewness estimates). Perhaps more importantly, we obtained the same results when restricting our analysis to place cells exhibiting positive skewness (Fig. 4); this argues against a ramp depolarization model since these cells receive greater excitation after the place field than before, but still display higher coupling to theta preceding place field entrance. Finally, a different class of models shows that phase precession can arise from network interactions rather than intrinsic cell properties^24, 25^; for example, a computational model has suggested that phase precession is due to asymmetric synaptic connections among place cells^27^. Whether network models can account for our results remains to be tested.

In summary, temporal coding by place cells has large asymmetry around the center of the rate-based place field. Our findings further revealed a three-stage dynamics for spatial coding through spike timing, in which place cells undergo phase coupling, sharp phase precession and phase decoupling as the animal traverses the place field. It should be noted that this three-stage classification is not based on a single variable but on the joint analysis of spiking phase and theta-coupling strength along space. The assessment of the spatial evolution of spike coupling to theta is the main novelty brought forward by our work and allowed for the new classification. While previous studies linked phase precession to changes in firing rate^8, 9, 11^, our results suggest that phase coupling and decoupling may be independent from the latter, and moreover happen at opposite theta phases. We conclude that place cells display temporal coding before rate coding along spatial trajectories, and hypothesize that the network mechanisms controlling spike timing and frequency differ.

## Materials and Methods

### Data set

We analyzed CA1 LFPs and spikes recorded with silicon-based multi-site probes from 3 male Long-Evans rats navigating in a 250-cm long linear track. This dataset is freely available online at the Collaborative Research in Computational Neuroscience website (https://crcns.org/), and was generously contributed by Gyorgy Buzsáki’s laboratory^28^. Detailed descriptions of the experimental procedures have been largely documented elsewhere^29–31^. Briefly, animals were implanted with a 4- or 8-shank silicon probe in the right dorsal hippocampus; shanks were 200 μm apart and had 8 recording sites^32^ (160 μm^2^, 1–3- MΩ impedance) separated by 20 μm. Signals were acquired on a 128-channel DataMax system at 20 kHz. Waveforms were sorted using KlustaKwik^33^ and manually adjusted with Kluster^34^. LFPs were obtained by down-sampling to 1250 Hz. Recording sites were identified *a posteriori* using histological data, electrophysiological benchmarks, and stereotaxic coordinates. Animals were video-recorded at 39.06 Hz; position on the linear track was estimated using two light-emitting diodes placed on the top of the head.

### Data analysis

All analyses were performed using built-in and custom written routines in MATLAB. For each shank and session, we analyzed the LFP from the channel with highest percentage of power in the theta range (5–12 Hz) in relation to all frequency range (0–625 Hz). Filtering was achieved by means of a finite impulse response filter from the EEGLAB toolbox^35^. The instantaneous phase was obtained using the analytical representation of the filtered signal based on the Hilbert transform, except in Supplementary Figure S3, in which we employed the linear interpolation method described in ref. 13.

### Place cells and place fields

We analyzed 100 sessions across the three animals. On each recording session, left and right runs were considered independently^36, 37^. We binned the linear track in 5-cm bins and calculated the spatial information per spike as described in ref. 38. Units with more than 1 bit of spatial information and with global firing rate higher than 0.3 Hz were considered putative place cells. We then computed continuous spatial firing rates by smoothing spike counts and spatial occupancy with a Gaussian kernel function (SD, 5 cm). Place fields were defined as contiguous regions (>20 cm) of firing rate above a threshold automatically set as half the average of the 50% highest firing rate bins (adapted from ref. 12). Place fields at the ends of the track (first and last 10 cm) were excluded from the analyses. Bimodal unidirectional place fields and bidirectional place fields (>50% field overlap between left and right runs) were considered as a single place field sample. Following these criteria, we obtained a total of 689 place cells and 1071 place fields.

### Phase coupling and normalized firing rate

To calculate spike-phase coupling strength as a function of space, we binned theta phase and relative distance to the place field center in non-overlapping bins of 20° and 0.1 place field length, respectively. At each space bin, spike-phase coupling was defined as a distance metric of the empirical spike-phase distribution from the uniform distribution, as previously described^39, 40^. Theta-phase coupling strength (TPC) was computed using the same metric but applied to the distribution of mean spiking phases. Therefore, while spike-phase coupling measures theta coupling of pooled spikes using data from all place fields, TPC estimates the consistency of the mean theta phase of spiking across different place fields. To delimit the region of significant TPC values in Fig. 3A,B, we generated a distribution of 1000 surrogate TPC curves, which were obtained by shifting the mean spiking phase within space bins by a random angle uniformly distributed between 0 and 2π. The statistical threshold was set as the 99^th^ percentile of the surrogate distribution. In Figs 3 and 5 and Supplementary Figures S3 and S4, the TPC curve was corrected by subtracting the mean surrogate curve. To compute the mean normalized firing rate (FR) curve, for each place field we divided the spatial firing rate by its maximum. TPC and FR curves were smoothed using a cubic spline before assessing the positions of peak and half maximum values; 99% confidence intervals for these parameters were obtained using 1000 random subsamples of 70% of place fields^41^.

### Data availability

The dataset analyzed in the current study is freely available in the CRCNS repository, https://crcns.org/data-sets/hc/hc-3. Analysis scripts can be obtained from the authors upon request.

## Electronic supplementary material

6 Supplementary Figures + Legends
