# Supplementary material for: Asymmetry of the temporal code for space by hippocampal place cells
Source: Sci Rep. 2017 Aug 17;7:8507. doi: 10.1038/s41598-017-08609-3 (PMC5561149; doi:10.1038/s41598-017-08609-3)
Supplement: Supplementary file 1 — 6 Supplementary Figures + Legends [file 41598_2017_8609_MOESM1_ESM.pdf]

Asymmetry of the temporal code for space by hippocampal place cells

Bryan C. Souza & Adriano B.L. Tort

Brain Institute, Federal University of Rio Grande do Norte, Natal, RN 59056-450, Brazil.

Supplementary Information

6 Supplementary Figures + Legends

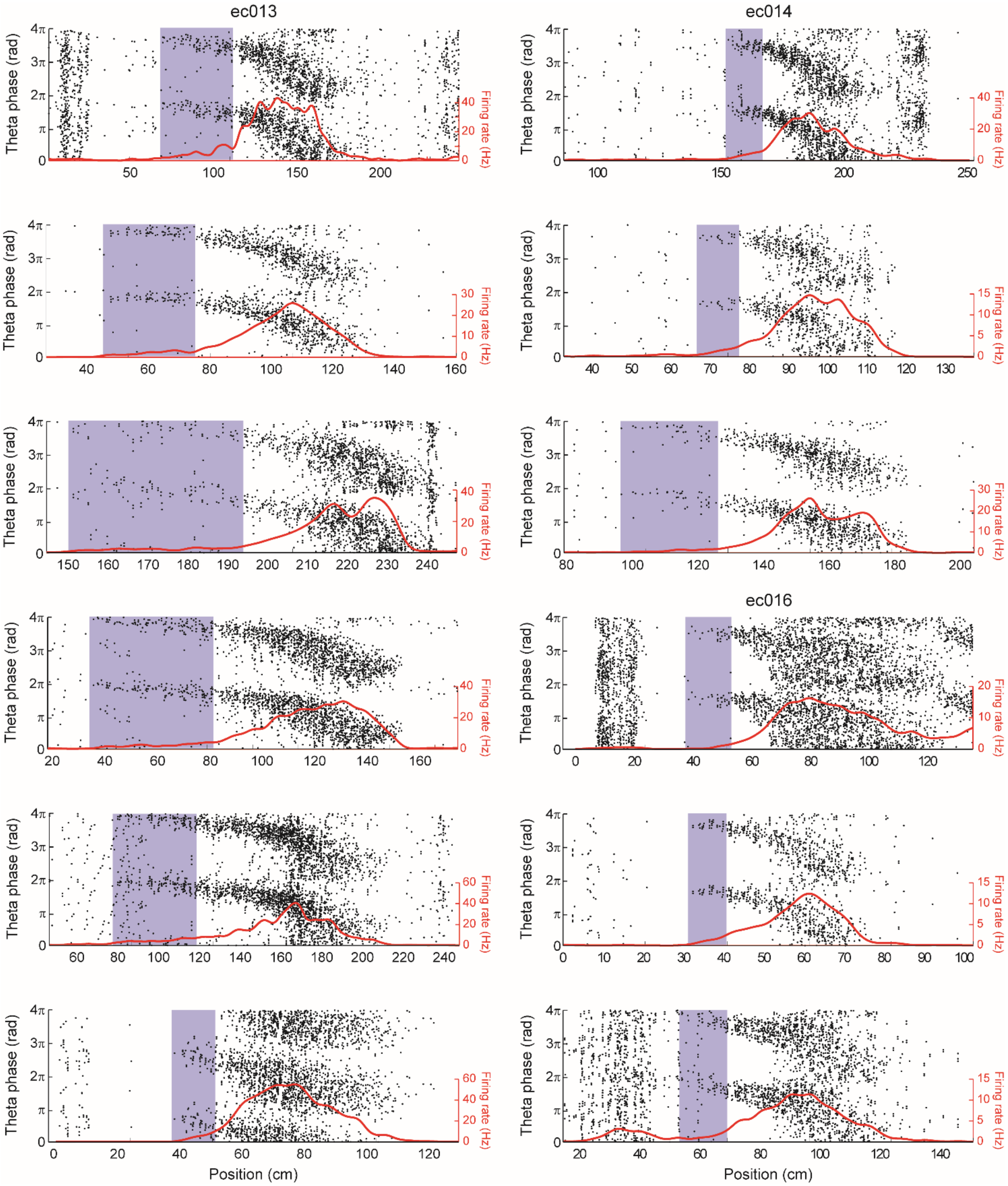

Supplementary Fig. S1. Examples of theta-phase coupling preceding major increases in firing rate in individual place cells. Blue shaded areas highlight locations where spikes align to a preferred theta phase before major increases in firing rate. Panels on the left column were obtained for example place cells recorded from rat ec013; top and bottom three panels on the right column were obtained for example cells from rats ec014 and ec016, respectively.

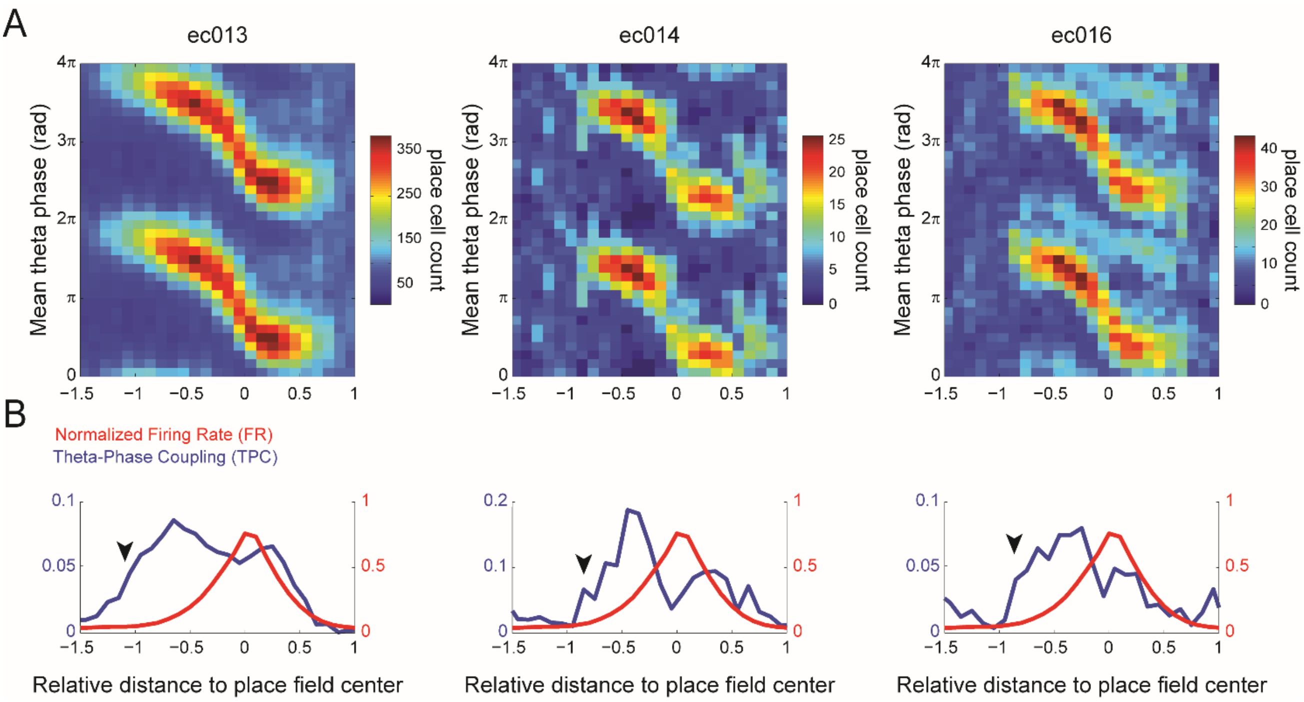

Supplementary Fig. S2. Asymmetry of temporal coding in individual animals. (A) Histograms of mean spiking phase, computed separately for each animal. At each position, individual place fields contribute with one count to the phase bin of their place cell's mean spiking. (B) Theta-phase coupling strength (TPC, blue) and mean normalized firing rate (FR, red) as functions of space. Arrowheads point to locations where place cells couple to theta before major changes in firing rate.

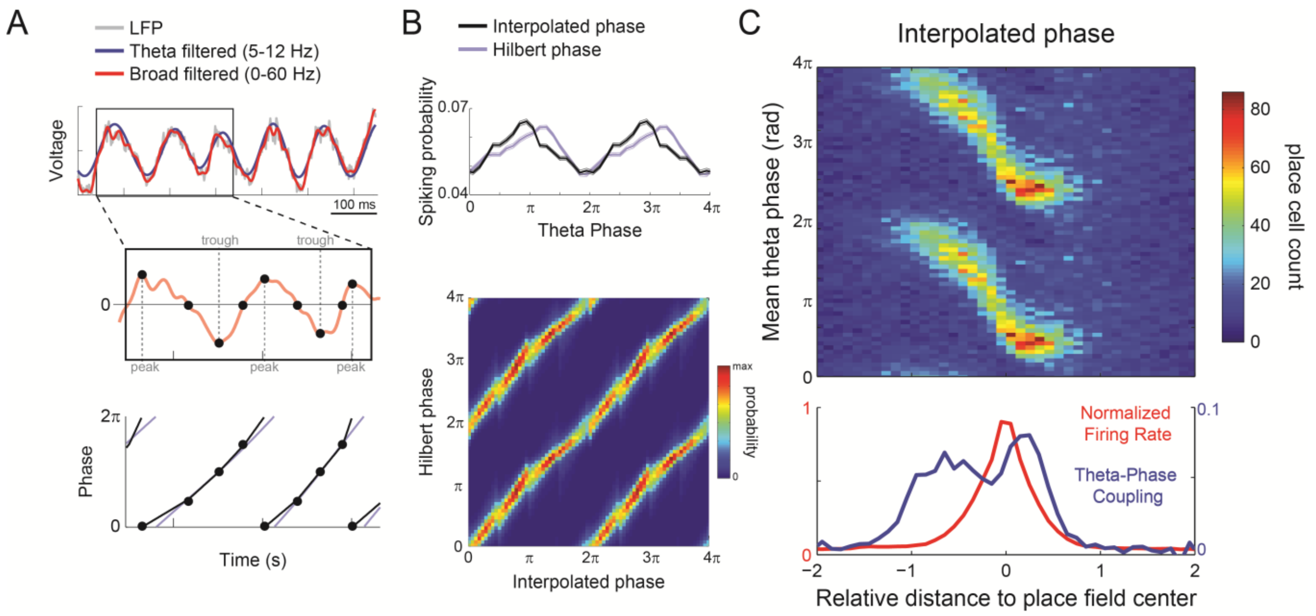

Supplementary Fig. S3. Temporal coding asymmetry persists when extracting theta phase by interpolation. (A) Estimation of theta phase by interpolation[13]. Peaks, troughs and zero-crossings (black dots) were detected from the broad-filtered LFP (0-60 Hz) and used to define the instantaneous phases corresponding to 0, π/2, π, and 3π/2; phase values between these points were estimated by linear interpolation (bottom; see ref. 13). (B) The top panel shows average (±SEM) spike-phase probability (across all place cells) using the interpolated phase (black) and the standard Hilbert-based phase extraction (blue). The bottom panel shows the joint probability of spikes. (C) The panels show the same as in Figure 3A,B, but for spatial dynamics of TPC computed using the theta phase estimated by interpolation. Notice similar results to the Hilbert-based phase extraction.

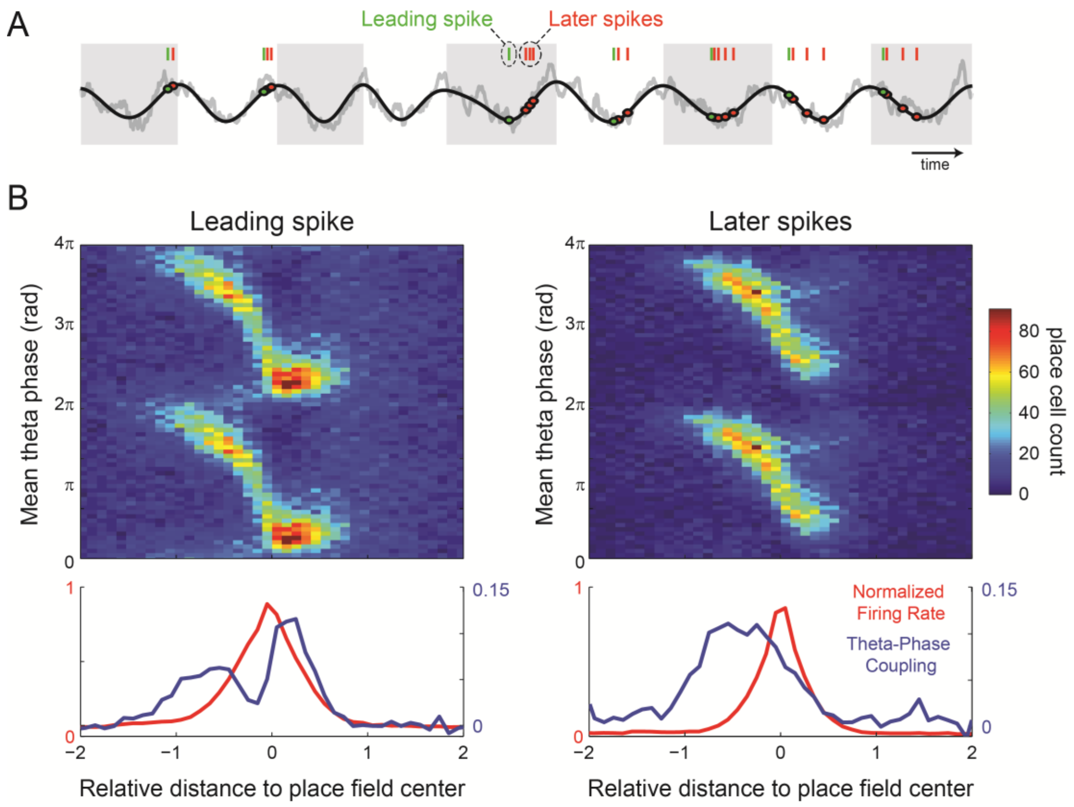

Supplementary Fig. S4. Temporal coding asymmetry occurs for both leading and later spikes within theta cycles. (A) Illustration of spike classification as "leading" or "later" spike per theta cycle. (B) The panels show the same as in Figure 3A,B, but for spatial dynamics of TPC computed separately for the leading spike (left) and later spikes (right). Notice that TPC increases before normalized firing rate in both cases and that phase-decoupling occurs even when only considering the leading spike.

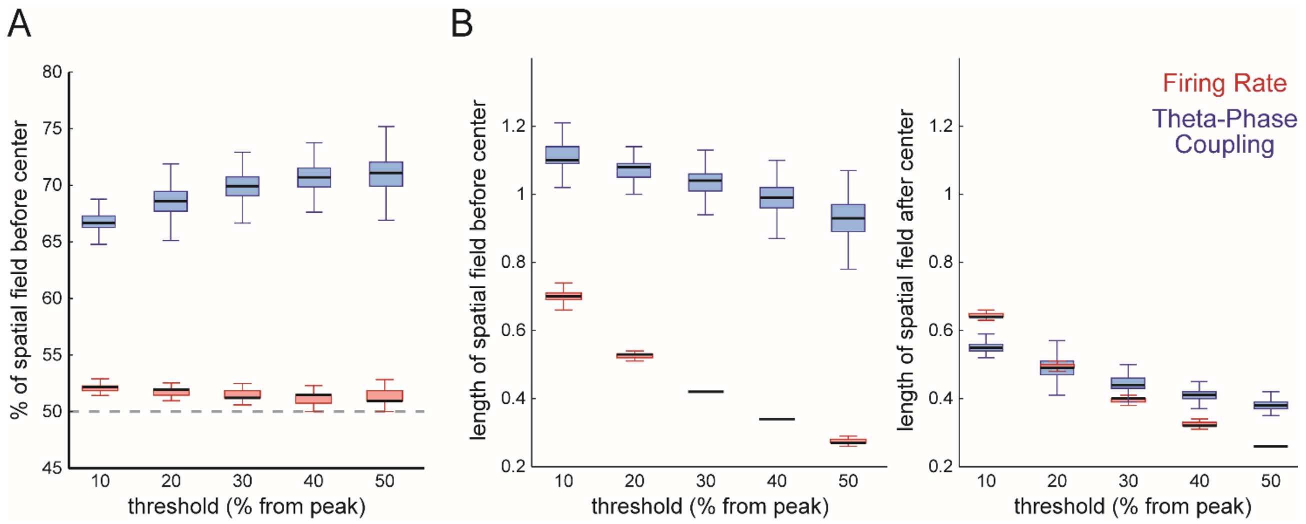

Supplementary Fig. S5. Temporal coding has greater asymmetry around the place field center than rate coding. (A) Percentage of the spatial receptive field before the place field center for TPC (blue) and FR (red) curves computed as in Figure 3F but for different thresholds (10, 20, 30, 40, and 50% of the TPC or FR peak value). Notice larger asymmetry for TPC spatial fields at all thresholds. (B) Length of the TPC and FR spatial fields before (left) and after (right) the place field center for different thresholds.

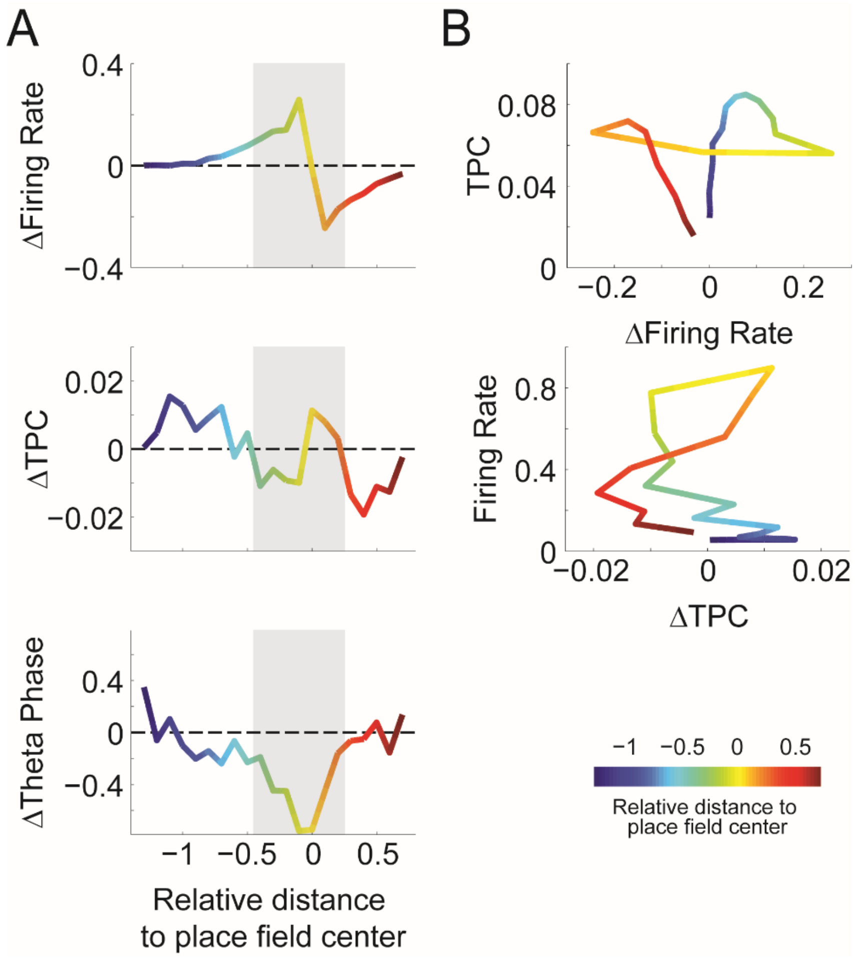

Supplementary Fig. S6. Spatial dynamics of the derivatives of firing rate, TPC and spiking theta phase. (A) Derivatives of the firing rate (top), TPC (middle) and spiking theta phase (bottom) traces shown in Figure 5. Gray shaded area separates place cell activity in three stages along space. (B) TPC values vs firing rate variations (top) and firing rate values vs TPC variations (bottom). Note in the top panel near-zero variation of firing rate while TPC greatly increases (blue colors). Colors code for the normalized distance to place field center.
